# Supplementary material for: Polyketide synthase gene clusters in the ericoid mycorrhizal fungus Oidiodendron maius: genome-wide prediction, expression profiling, and functional characterization of knockout mutants
Source: Front Plant Sci. 2026 Sep 16;17:1924430. doi: 10.3389/fpls.2026.1924430 (PMC13623903; doi:10.3389/fpls.2026.1924430)
Supplement: Supplementary file 1 [file SupplementaryFile1.zip › Supplementary Materials/Supplementary Folder 1.zip!/Oidma1_AssemblyScaffolds_Repeatmasked/knownclusterblast/cluster41/jgi.p_Oidma1_133487_mibig_hits.html]

| MIBiG Protein | Description | MIBiG Cluster | MiBiG Product | % ID | % Coverage | BLAST Score | E-value |
| --- | --- | --- | --- | --- | --- | --- | --- |
| AIG62140.1 | ABC\_transporter | BGC0000120 | Patulin\_biosynthetic\_gene\_cluster | 34 | 38.7724550898 | 140 | 7.9e-33 |
| CAB01940.1 | ExpD1 | BGC0000801 | Galactoglucan\_biosynthetic\_gene\_cluster | 31 | 41.0179640719 | 107 | 5.7e-23 |
| AAP85350.1 | ATP-binding\_protein | BGC0000233 | Hedamycin\_biosynthetic\_gene\_cluster | 32 | 33.8323353293 | 103 | 8.2e-22 |
| AAX31540.1 | putative\_ABC\_transporter\_ATP-binding\_protein | BGC0000336 | Daptomycin\_biosynthetic\_gene\_cluster | 31 | 34.5808383234 | 97 | 7.7e-20 |
| ADK32548.1 | putative\_ABC\_transporter\_ATP-binding\_protein | BGC0000529 | Microbisporicin\_biosynthetic\_gene\_cluster | 28 | 38.622754491 | 96 | 1e-19 |
| ABS75252.1 | putative\_ABC-transporter\_ATP-binding\_protein | BGC0000616 | Amylocyclicin\_biosynthetic\_gene\_cluster | 32 | 32.1856287425 | 96 | 1e-19 |
| ACN64822.1 | PokABC1 | BGC0001061 | Polyketomycin\_biosynthetic\_gene\_cluster | 32 | 34.2814371257 | 96 | 1e-19 |
| AKD43524.1 | ABC\_transporter | BGC0001409 | Dutomycin\_biosynthetic\_gene\_cluster | 31 | 34.2814371257 | 95 | 2.2e-19 |
| AHI59113.1 | ATPase | BGC0001005 | Locillomycin\_biosynthetic\_gene\_cluster | 31 | 31.1377245509 | 95 | 2.9e-19 |
| EHQ60564.1 | ABC\_transporter | BGC0001356 | Paeninodin\_biosynthetic\_gene\_cluster | 32 | 34.880239521 | 95 | 2.9e-19 |
| ACB47076.1 | DynT5 | BGC0001060 | Dynemicin\_biosynthetic\_gene\_cluster | 30 | 33.2335329341 | 95 | 2.9e-19 |
| ABW00319.1 | daunorubicin\_resistance\_ABC\_transporter\_ATPase\_subunit | BGC0000333 | Cyclomarin\_biosynthetic\_gene\_cluster | 29 | 35.4790419162 | 94 | 5e-19 |
| ALD83698.1 | ABC\_transporter\_ATP\_binding\_protein | BGC0001299 | Chlorotonil\_biosynthetic\_gene\_cluster | 35 | 30.9880239521 | 94 | 6.5e-19 |
| AAK73498.1 | AmphG | BGC0000015 | Amphotericin\_biosynthetic\_gene\_cluster | 29 | 34.7305389222 | 94 | 6.5e-19 |
| AAU39356.1 | putative\_spermidine/putrescine\_ABC\_transporter\_ATP-binding\_protein | BGC0000381 | Lichenysin\_biosynthetic\_gene\_cluster | 33 | 30.8383233533 | 93 | 8.5e-19 |
| AFP87531.1 | ABC\_transporter | BGC0001159 | Marinopyrrole\_biosynthetic\_gene\_cluster | 31 | 31.7365269461 | 93 | 8.5e-19 |
| AAU34217.1 | ABC\_transporter\_MppX | BGC0000388 | Mannopeptimycin\_biosynthetic\_gene\_cluster | 32 | 28.1437125749 | 93 | 1.1e-18 |
| AAG32068.1 | putative\_ATP\_binding\_protein | BGC0000026 | Avilamycin\_A\_biosynthetic\_gene\_cluster | 31 | 32.7844311377 | 92 | 1.4e-18 |
| ALG65297.1 | Cal39 | BGC0001297 | WS9326\_biosynthetic\_gene\_cluster | 31 | 32.0359281437 | 92 | 1.4e-18 |
| BAH04156.1 | putative\_ABC\_transporter | BGC0000450 | Triostin\_A\_biosynthetic\_gene\_cluster | 31 | 30.6886227545 | 92 | 2.5e-18 |
| BAJ05895.1 | putative\_ABC\_multidrug\_resistance\_transporter | BGC0000872 | A-90289\_biosynthetic\_gene\_cluster | 30 | 40.8682634731 | 91 | 4.2e-18 |
| AHX24693.1 | ABC\_transporter\_ATP-binding\_protein | BGC0000200 | Arixanthomycin\_biosynthetic\_gene\_cluster | 31 | 33.5329341317 | 91 | 5.5e-18 |
| AAG32538.1 | SalT | BGC0000548 | Salivaricin\_A\_biosynthetic\_gene\_cluster | 27 | 35.628742515 | 90 | 9.4e-18 |
| BAO66521.1 | ABC\_transporter | BGC0000042 | Cremimycin\_biosynthetic\_gene\_cluster | 31 | 33.8323353293 | 90 | 9.4e-18 |
| AJW76714.1 | DsaL | BGC0001196 | Desotamide\_biosynthetic\_gene\_cluster | 33 | 31.5868263473 | 89 | 1.6e-17 |
| AIS24846.1 | Dst20 | BGC0001147 | Distamycin\_biosynthetic\_gene\_cluster | 28 | 39.0718562874 | 89 | 1.6e-17 |
| AAX98195.1 | ABC\_transporter | BGC0000052 | ECO-02301\_biosynthetic\_gene\_cluster | 33 | 26.9461077844 | 89 | 2.1e-17 |
| AAF71763.1 | NysG | BGC0000115 | Nystatin\_biosynthetic\_gene\_cluster | 28 | 34.7305389222 | 88 | 3.6e-17 |
| AHL46714.1 | ABc\_transporter\_protein | BGC0001179 | Rishirilide\_B\_biosynthetic\_gene\_cluster | 29 | 35.4790419162 | 88 | 3.6e-17 |
| BAB78441.1 |  | BGC0000555 | Staphylococcin\_C55\_alpha\_/\_staphylococcin\_C55\_beta\_biosynthetic\_gene | 29 | 34.880239521 | 86 | 1e-16 |
| CAJ88181.1 | putative\_ABC\_transporter,\_ATP-binding\_subunit | BGC0000151 | Stambomycin\_biosynthetic\_gene\_cluster | 31 | 28.8922155689 | 86 | 1.4e-16 |
| ABA00875.1 | NsuF | BGC0000537 | Nisin\_U\_biosynthetic\_gene\_cluster | 28 | 32.3353293413 | 86 | 1.8e-16 |
| CAA90021.1 | PepT | BGC0000543 | Pep5\_biosynthetic\_gene\_cluster | 29 | 30.9880239521 | 86 | 1.8e-16 |
| CAC20925.1 | PimB\_protein | BGC0000125 | Pimaricin\_biosynthetic\_gene\_cluster | 28 | 34.7305389222 | 84 | 5.1e-16 |
| AFN69431.1 | ElxT | BGC0000509 | Epilancin\_15X\_biosynthetic\_gene\_cluster | 28 | 32.1856287425 | 84 | 5.1e-16 |
| AGS77319.1 | ABC\_transporter | BGC0001178 | UK-68,597\_biosynthetic\_gene\_cluster | 27 | 40.4191616766 | 84 | 6.7e-16 |
| BAI63276.1 | putative\_ABC\_transporter | BGC0000434 | SW-163\_biosynthetic\_gene\_cluster | 30 | 30.6886227545 | 84 | 6.7e-16 |
| AEK75511.1 | ATP-binding\_ABC\_peptide\_transporter | BGC0000001 | Abyssomicin\_biosynthetic\_gene\_cluster | 27 | 35.628742515 | 84 | 6.7e-16 |
| AIG79244.1 | Conserved\_putative\_membrane\_protein | BGC0000419 | Ristomycin\_A\_biosynthetic\_gene\_cluster | 31 | 30.3892215569 | 84 | 6.7e-16 |
| ADX66465.1 | ScnB | BGC0000108 | Natamycin\_biosynthetic\_gene\_cluster | 28 | 34.7305389222 | 83 | 8.8e-16 |
| CAA11793.1 | PCZA363.2 | BGC0000322 | Chloroeremomycin\_biosynthetic\_gene\_cluster | 29 | 30.3892215569 | 83 | 1.1e-15 |
| WP\_028678143.1 | daunorubicin\_resistance\_protein\_DrrA\_family\_ABC\_transporter\_ATP-binding\_protein | BGC0001228 | Retimycin\_biosynthetic\_gene\_cluster | 31 | 25.4491017964 | 83 | 1.1e-15 |
| AIE77056.1 | ABC-transporter | BGC0000418 | Ristocetin\_biosynthetic\_gene\_cluster | 30 | 30.3892215569 | 83 | 1.1e-15 |
| ADC96668.1 | ABC\_transporter | BGC0001076 | Liposidomycin\_biosynthetic\_gene\_cluster | 31 | 34.2814371257 | 81 | 4.4e-15 |
| CCM44327.1 | putative\_ABC\_transporter\_ATP-binding\_protein | BGC0001056 | Zeamine\_biosynthetic\_gene\_cluster | 30 | 29.4910179641 | 80 | 7.4e-15 |
| AEI58864.1 | ABC\_transporter | BGC0000455 | Vancomycin\_biosynthetic\_gene\_cluster | 30 | 30.9880239521 | 80 | 9.7e-15 |
| AAZ23072.1 | hypothetical\_protein | BGC0000291 | A54145\_biosynthetic\_gene\_cluster | 30 | 28.5928143713 | 80 | 9.7e-15 |
| AAM80540.1 | StaU | BGC0000290 | A47934\_biosynthetic\_gene\_cluster | 29 | 30.8383233533 | 79 | 1.3e-14 |
| ADO17962.1 | CclG | BGC0000487 | Carnocyclin\_biosynthetic\_gene\_cluster | 31 | 28.2934131737 | 79 | 1.3e-14 |
| ALK27913.1 | putative\_ABC\_transporter | BGC0001233 | Feglymycin\_biosynthetic\_gene\_cluster | 25 | 41.9161676647 | 79 | 2.2e-14 |
| CCM44340.1 | putative\_ABC\_transporter\_ATP-binding\_protein | BGC0001056 | Zeamine\_biosynthetic\_gene\_cluster | 31 | 30.0898203593 | 78 | 2.8e-14 |
| ABD65953.1 | ABC\_transporter | BGC0000341 | Enduracidin\_biosynthetic\_gene\_cluster | 28 | 31.5868263473 | 78 | 3.7e-14 |
| CAD91219.1 | putative\_ABC\_transporter,\_Dbv24 | BGC0000289 | A40926\_biosynthetic\_gene\_cluster | 29 | 32.4850299401 | 77 | 4.8e-14 |
| AAY28215.1 | unknown | BGC0000074 | Herbimycin\_biosynthetic\_gene\_cluster | 30 | 34.4311377246 | 77 | 6.3e-14 |
| AAN85531.1 | ABC\_transporter\_component,\_ATP\_hydrolase | BGC0001101 | Leinamycin\_biosynthetic\_gene\_cluster | 27 | 33.8323353293 | 77 | 8.2e-14 |
| AAX31526.1 | putative\_ABC\_transporter | BGC0000336 | Daptomycin\_biosynthetic\_gene\_cluster | 32 | 36.9760479042 | 76 | 1.1e-13 |
| AAK81823.1 | ABC\_transporter | BGC0000326 | Complestatin\_biosynthetic\_gene\_cluster | 27 | 35.0299401198 | 76 | 1.1e-13 |
| ACQ63630.1 | ABC\_multidrug\_resistance\_transporter | BGC0000875 | Caprazamycin\_biosynthetic\_gene\_cluster | 31 | 35.1796407186 | 76 | 1.1e-13 |
| AHA81996.1 | Arm27 | BGC0000199 | Arimetamycin\_biosynthetic\_gene\_cluster | 29 | 32.3353293413 | 76 | 1.4e-13 |
| EEM14530.1 | hypothetical\_protein | BGC0001210 | Pseudomycoicidin\_biosynthetic\_gene\_cluster | 29 | 31.8862275449 | 76 | 1.4e-13 |
| AAX31547.1 | putative\_ABC\_transporter\_ATP-binding\_protein | BGC0000336 | Daptomycin\_biosynthetic\_gene\_cluster | 31 | 32.9341317365 | 76 | 1.8e-13 |
| AAM54082.1 | ABC\_transporter | BGC0000020 | Ansamitocin\_biosynthetic\_gene\_cluster | 27 | 40.119760479 | 76 | 1.8e-13 |
| CAG15016.1 | ABC\_transporter | BGC0000441 | Teicoplanin\_biosynthetic\_gene\_cluster | 27 | 32.4850299401 | 75 | 2.4e-13 |
| CAE53357.1 | ABC\_transporter | BGC0000440 | Teicoplanin\_biosynthetic\_gene\_cluster | 27 | 32.4850299401 | 75 | 2.4e-13 |
| ALD83689.1 | ATP\_binding\_protein | BGC0001300 | Anthracimycin\_biosynthetic\_gene\_cluster | 32 | 25.8982035928 | 75 | 3.1e-13 |
| CBL93714.1 | ABC\_multidrug\_transporter | BGC0000360 | GE81112\_biosynthetic\_gene\_cluster | 30 | 32.4850299401 | 74 | 4.1e-13 |
| ALE27499.1 | ABC\_transporter | BGC0001292 | A33853\_biosynthetic\_gene\_cluster | 26 | 35.0299401198 | 74 | 6.9e-13 |
| AAP03990.1 | putative\_microcin\_L\_transport\_protein | BGC0000588 | Microcin\_L\_biosynthetic\_gene\_cluster | 25 | 34.880239521 | 73 | 9.1e-13 |
| AFD30963.1 | CrmT2 | BGC0000966 | Caerulomycin\_A\_biosynthetic\_gene\_cluster | 30 | 31.4371257485 | 73 | 1.2e-12 |
| AAD56148.1 | MutT | BGC0000533 | Mutacin\_III\_biosynthetic\_gene\_cluster | 30 | 25.5988023952 | 72 | 1.5e-12 |
| CAE55703.1 | probable\_microcin\_H47\_secretion\_ATP-binding\_protein\_mchF | BGC0000589 | Microcin\_M\_biosynthetic\_gene\_cluster | 25 | 34.880239521 | 72 | 1.5e-12 |
| CAJ44959.1 | MchF\_protein | BGC0000587 | Microcin\_H47\_biosynthetic\_gene\_cluster | 25 | 34.880239521 | 72 | 1.5e-12 |
| CAG34715.1 | putative\_ABC\_transporter | BGC0000713 | Ribostamycin\_biosynthetic\_gene\_cluster | 28 | 35.7784431138 | 72 | 1.5e-12 |
| BAI23328.1 | putative\_ABC-transporter | BGC0000949 | A-500359s\_biosynthetic\_gene\_cluster | 28 | 33.9820359281 | 72 | 2e-12 |
| CAD15510.1 | probable\_composite\_atp-binding\_transmembrane\_abc\_transporter\_protein | BGC0001014 | Micacocidin\_biosynthetic\_gene\_cluster | 31 | 35.7784431138 | 72 | 2e-12 |
| ADK32562.1 | putative\_lantibiotic\_ABC\_transporter\_ATP-binding\_protein | BGC0000529 | Microbisporicin\_biosynthetic\_gene\_cluster | 28 | 31.1377245509 | 72 | 2.6e-12 |
| CTQ34884.1 | AtcG;\_putative\_ABC\_transporter | BGC0001301 | Anthracimycin\_biosynthetic\_gene\_cluster | 34 | 25.8982035928 | 71 | 4.5e-12 |
| AFD30962.1 | CrmT3 | BGC0000966 | Caerulomycin\_A\_biosynthetic\_gene\_cluster | 28 | 35.1796407186 | 71 | 5.9e-12 |
| CAC33840.1 | hypothetical\_protein | BGC0000523 | Lactocin\_S\_biosynthetic\_gene\_cluster | 25 | 33.5329341317 | 71 | 5.9e-12 |
| CAP20371.1 | ABC\_transporter\_ATP-binding\_membrane\_protein | BGC0000347 | Equibactin\_biosynthetic\_gene\_cluster | 29 | 32.6347305389 | 70 | 7.7e-12 |
| AAD28497.1 | McjD | BGC0000581 | Microcin\_J25\_biosynthetic\_gene\_cluster | 29 | 32.4850299401 | 70 | 1e-11 |
| AAL08400.1 | MceG | BGC0000586 | Microcin\_E492\_biosynthetic\_gene\_cluster | 25 | 33.0838323353 | 68 | 2.9e-11 |
| ADH04633.1 | unknown | BGC0001051 | Thuggacin\_biosynthetic\_gene\_cluster | 29 | 35.9281437126 | 68 | 3.8e-11 |
| AHN91916.1 | ABC\_transporter | BGC0000340 | Echosides\_biosynthetic\_gene\_cluster | 26 | 36.9760479042 | 67 | 6.5e-11 |
| ctg1\_orf5 |  | BGC0000321 | Chaetocin\_biosynthetic\_gene\_cluster | 27 | 32.7844311377 | 67 | 6.5e-11 |
| BAH43868.1 | probable\_ABC\_transporter\_ATP\_binding\_protein | BGC0000367 | Gramicidin\_biosynthetic\_gene\_cluster | 27 | 27.8443113772 | 67 | 6.5e-11 |
| AAN85547.1 | ABC\_transporter | BGC0001101 | Leinamycin\_biosynthetic\_gene\_cluster | 28 | 34.2814371257 | 67 | 8.5e-11 |
| CAA11515.1 | colicin\_V\_exporter\_protein | BGC0000584 | Colicin\_V\_biosynthetic\_gene\_cluster | 26 | 30.9880239521 | 67 | 8.5e-11 |
| BAJ19059.1 | putative\_ABC\_transporter | BGC0000288 | A-503083\_biosynthetic\_gene\_cluster | 25 | 33.5329341317 | 66 | 1.1e-10 |
| ABV83219.1 | CppH | BGC0000116 | Nystatin-like\_Pseudonocardia\_polyene\_biosynthetic\_gene\_cluster | 25 | 44.0119760479 | 66 | 1.4e-10 |
| CAP20370.1 | ABC\_transporter,\_ATP-binding\_membrane\_protein | BGC0000347 | Equibactin\_biosynthetic\_gene\_cluster | 27 | 30.8383233533 | 66 | 1.4e-10 |
| CAX48975.1 | ABC-transporter | BGC0000519 | Labyrinthopeptin\_A1,A3\_/\_labyrinthopeptin\_A2\_biosynthetic\_gene | 28 | 31.1377245509 | 66 | 1.4e-10 |
| BAE95566.1 | putative\_oligopeptide\_ABC\_transporter\_ATP-binding\_protein | BGC0000703 | Kanamycin\_biosynthetic\_gene\_cluster | 27 | 35.9281437126 | 65 | 2.5e-10 |
| CAA07371.1 | StrW | BGC0000690 | 5'-hydroxystreptomycin\_biosynthetic\_gene\_cluster | 27 | 33.3832335329 | 65 | 2.5e-10 |
| NP\_230428.1 | ferric\_vibriobactin\_ABC\_transporter\_ATP-binding\_protein | BGC0000945 | Vibriobactin\_biosynthetic\_gene\_cluster | 26 | 31.8862275449 | 65 | 2.5e-10 |
| CAA12529.1 | ExsA\_protein | BGC0000802 | Succinoglycan\_biosynthetic\_gene\_cluster | 27 | 34.880239521 | 65 | 2.5e-10 |
| CAH58693.1 | putative\_exporter | BGC0000711 | Neomycin\_biosynthetic\_gene\_cluster | 25 | 34.1317365269 | 65 | 3.2e-10 |
| CAH05098.1 | ABC\_transport\_protein | BGC0000710 | Neomycin\_biosynthetic\_gene\_cluster | 25 | 34.1317365269 | 65 | 3.2e-10 |
| AAK01464.1 | ABC\_transporter | BGC0000412 | Pyochelin\_biosynthetic\_gene\_cluster | 27 | 33.8323353293 | 65 | 3.2e-10 |
| CAF33315.1 | putative\_ABC\_transporter,\_ATP-binding\_protein,\_NeoU | BGC0000709 | Neomycin\_biosynthetic\_gene\_cluster | 25 | 34.1317365269 | 65 | 3.2e-10 |
| BAE46917.1 | goadsporin\_biosynthetic\_protein | BGC0000565 | Goadsporin\_biosynthetic\_gene\_cluster | 26 | 33.8323353293 | 64 | 4.2e-10 |
| CAF31837.1 | putative\_hygromycin\_B\_exporter | BGC0000699 | Hygromycin\_B\_biosynthetic\_gene\_cluster | 26 | 34.4311377246 | 64 | 4.2e-10 |
| BAC87910.1 | probable\_ABC\_transporter | BGC0000294 | Acinetobactin\_biosynthetic\_gene\_cluster | 34 | 26.9461077844 | 64 | 4.2e-10 |
| ABW91161.1 | exporter/resistance\_protein | BGC0000019 | Angolamycin\_biosynthetic\_gene\_cluster | 26 | 36.8263473054 | 62 | 2.7e-09 |
| ADU86007.1 | putative\_ABC\_transporter-related\_protein | BGC0000165 | Tiacumicin\_B\_biosynthetic\_gene\_cluster | 30 | 24.251497006 | 61 | 6.1e-09 |
| BAP82652.1 | ABC\_transporter | BGC0001148 | Pheganomycin\_biosynthetic\_gene\_cluster | 27 | 32.7844311377 | 60 | 1e-08 |
| CAH94306.1 | putative\_ABC\_transporter\_StrW | BGC0000717 | Streptomycin\_biosynthetic\_gene\_cluster | 27 | 34.880239521 | 59 | 1.4e-08 |
| BAB78444.1 |  | BGC0000555 | Staphylococcin\_C55\_alpha\_/\_staphylococcin\_C55\_beta\_biosynthetic\_gene | 25 | 30.9880239521 | 59 | 1.4e-08 |
| BAG22745.1 | putative\_ABC-type\_multidrug\_transport\_system\_ATPase\_and\_permease\_component | BGC0000724 | Streptomycin\_biosynthetic\_gene\_cluster | 27 | 34.880239521 | 59 | 1.4e-08 |
| CAG38692.1 | transport\_protein | BGC0000708 | Lividomycin\_biosynthetic\_gene\_cluster | 29 | 31.1377245509 | 58 | 3e-08 |
| ADZ45322.1 | ABC\_transporter-like\_protein | BGC0001020 | Muraymycin\_biosynthetic\_gene\_cluster | 25 | 31.8862275449 | 58 | 3.9e-08 |
| AAT03890.1 | ABC\_transporter,\_ATP-binding\_protein | BGC0001171 | Listeriolysin\_S\_biosynthetic\_gene\_cluster | 26 | 30.8383233533 | 56 | 1.1e-07 |
| ABX00624.1 | LmrC | BGC0000907 | Lincomycin\_biosynthetic\_gene\_cluster | 30 | 25.748502994 | 55 | 3.3e-07 |
| BAE95543.1 | ABC\_transporter\_ATP-binding\_protein | BGC0000703 | Kanamycin\_biosynthetic\_gene\_cluster | 27 | 32.9341317365 | 55 | 3.3e-07 |
| CCA89322.1 | hypothetical\_protein | BGC0001111 | Rhizopodins\_biosynthetic\_gene\_cluster | 23 | 31.4371257485 | 55 | 3.3e-07 |
| CAA19963.1 | putative\_ABC\_transporter | BGC0000551 | SapB\_biosynthetic\_gene\_cluster | 28 | 29.7904191617 | 54 | 5.7e-07 |
| CAM02315.1 | putative\_ABC\_transporter\_transmembrane | BGC0000349 | Erythrochelin\_biosynthetic\_gene\_cluster | 25 | 32.7844311377 | 52 | 2.8e-06 |
| AHZ20760.1 | ABC\_transporter\_transmembrane\_domain/ATP-binding\_domain\_protein | BGC0000369 | Hassallidins\_biosynthetic\_gene\_cluster | 27 | 29.9401197605 | 51 | 3.7e-06 |
